# The Beneficial Effect of Vanillic Acid on Ulcerative Colitis

**DOI:** 10.3390/molecules15107208

**Published:** 2010-10-19

**Authors:** Su-Jin Kim, Min-Cheol Kim, Jae-Young Um, Seung-Heon Hong

**Affiliations:** 1 Department of Pharmacology, College of Oriental Medicine, Institute of Oriental Medicine, Kyung Hee University; Dongdaemun-Gu, Seoul 130-701, Korea; E-Mails: ksj1009@khu.ac.kr (S.-J.K); jyum@khu.ac.kr (J.-Y.U); 2 Wonkwang Oriental Medicines Research Institute, Department of Oriental Pharmacy, College of Pharmacy, Wonkwang University, Iksan, Jeonbuk 570-749, Korea; E-Mail: mchol@naver.com (M.-C.K)

**Keywords:** vanillic acid, dextran sulfate sodium, ulcerative colitis, cyclooxygenase-2, nuclear factor-κB p65, interleukin-6

## Abstract

Vanillic acid, an oxidized form of vanillin, is a benzoic acid derivative used as a flavoring agent. The objective of this study was to determine whether vanillic acid has beneficial effects against dextran sulfate sodium (DSS)-induced ulcerative colitis. Our results showed that vanillic acid reduced the severity of the clinical signs of DSS-induced colitis, including weight loss and shortening of colon length, and the disease activity index. The results of this study showed that vanillic acid significantly suppressed the expression of cyclooxygenase-2 and the activation of transcription nuclear factor-κB p65 in DSS-treated colon tissues. In addition, we observed that the plasma levels of interleukin (IL)-6 were higher in the DSS-treated group than in the control group, but these increased levels were reduced by the administration of vanillic acid. Taken together, these findings suggest that vanillic acid has a beneficial effect on DSS-induced ulcerative colitis, thereby indicating its usefulness in the regulation of chronic intestinal inflammation.

## 1. Introduction 

Ulcerative colitis (UC) is a typical inflammatory intestinal disease belonging to the class of inflammatory bowel diseases (IBD) [1,2]. The pathogenesis of UC is believed to involve the interaction of genetic, immune, and environmental factors [3]. UC is associated with intestinal inflammation and often results in weight loss, diarrhea accompanied by blood and mucus, fever, gastric dysmotility, and shortening of the colon [4]. Pathologic hallmarks of UC include ulceration of the mucosa, blunting and loss of crypts, and infiltration of inflammatory cells [5]. These pathologic changes frequently cause epithelial dysplasia and DNA damage with microsatellite instability [6]. Additionally, prolonged and chronic UC may progress to a colorectal cancer. Thus, to develop measures to prevent cancer development in UC patients, it is necessary to understand the pathogenesis of UC at the molecular and cellular levels.

Current studies on UC have reported that proinflammatory cytokines are involved in the initiation of the inflammatory response in colitis. It was reported that mucosa from patients with UC shows increased expression of interleukin (IL)-1 and IL-6, which are believed to play an integral role in the pathogenesis of UC [7]. Hence, there is currently a strong interest in agents that can block the generation or activities of inflammatory cytokines. Cyclooxygenases (COXs) have been implicated in a number of physiological events, including the progression of inflammation, immunomodulation, and transmission of pain. Two COX isoenzymes have been identified. COX-1 is a constitutive enzyme that is required for the formation of prostaglandins (PGs) protecting the stomach and kidney from damage. COX-2, which is normally expressed at very low levels, can be rapidly induced by a variety of stimuli such as cytokines, growth factors, hormones, and carcinogens and is believed to be responsible for the production of prostaglandins associated with mediation of inflammation. COX-2 expression is reported to be increased in the inflamed mucosa of patients with UC [8,9].

Nuclear factor (NF)-κB performs a crucial function in the expression of many genes involved in immune and inflammatory responses. Active NF-κB-IκB complexes are sequestered in the cytoplasm [10,11]. On induction by a variety of stimuli, NF-κB translocates into the nucleus where it can bind to specific DNA sequences located in the promoter regions of target genes and activate gene transcription; thus, by controlling the transcription of inflammatory cytokine genes, it plays a pivotal role in the regulation of immune and inflammatory responses. Increased DNA-binding activity of NF-κB associated with the secretion of high levels of IL-1 and IL-6 has been reported in macrophages of UC patients. In lieu of these properties, NF-κB has been recognized as an ideal target for molecular therapies employed to treat inflammatory diseases [12].

Generally, UC is characterized clinically by acute exacerbation, and corticosteroids have proven to be effective in bringing about clinical remission [13]. However, in some severe cases of relapse, corticosteroids may not be effective even when administered in high doses (more than 1 mg·kg^-1^·day^-1^ orally or intravenously). In addition, the long-term use of corticosteroids is often associated with serious adverse effects such as hormonal disturbances, peptic ulcers, liver dysfunction, and psychological problems. The appearance of these adverse effects may necessitate discontinuation of corticosteroid treatment, thereby resulting in acute exacerbation. Therefore, an alternative treatment for active UC is necessary to eliminate the clinical problems associated with corticosteroid therapy. 

Vanillic acid is a benzoic acid derivative used as a flavoring agent. It is an oxidized form of vanillin produced during the conversion of vanillin to ferulic acid [14,15]. The highest quantity of vanillic acid in plants has been found in the roots of *Angelica sinensis*, which is used in traditional Chinese medicine [16]. Various studies have provided evidence of the effectiveness of vanillic acid in the management of immune or inflammatory responses. For instance, vanillic acid enhanced the activity of human lymphocyte proliferation and secretion of interferon-gamma in human peripheral blood mononuclear cells [17]. Another study has shown that vanillic acid has a hepatoprotective effect through its suppressive action on immune-mediated liver inflammation in concanavalin A-induced liver injury [18]. However, it remains to be determined whether vanillic acid has an anti-colitic effect.

Another study reported that mice with dextran sodium sulfate (DSS)-induced colitis exhibit phenotypical characteristics similar to human acute and chronic UC [19]. The aim of this study was to examine the effects of vanillic acid on the pathogenesis of DSS-induced colitis. The specific aims of the study were as follows: (1) to assess the effect of vanillic acid on the clinical signs of UC, including body weight, colon length, severity of diarrhea, and extent of occult/gross bleeding, and (2) to investigate the effect of vanillic acid on the expression of inflammation-related genes in DSS-treated colon tissues. 

## 2. Results and Discussion 

In the present study, we investigated the effect of vanillic acid on DSS-induced experimental colitis in mice models exhibiting a phenotype similar to human acute and chronic UC.

### 2.1. The effect of vanillic acid on clinical signs in DSS-induced colitis 

**Figure 1:**
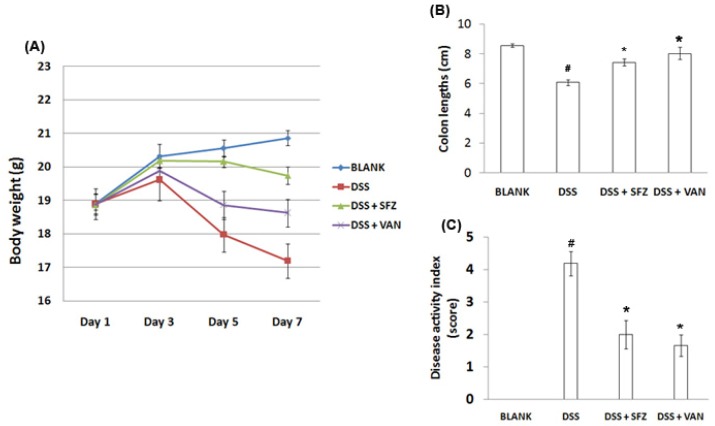
Effect of vanillic acid on the clinical signs in DSS-induced colitis. Experimental colitis in mice was induced by 7-day treatment with 5% DSS dissolved in drinking water. Vanillic acid was administered orally at doses of 200 mg/kg once a day for seven days before DSS treatment. (A) Body weight of the mice was measured. (B) The colons were removed on day 7 after DSS treatment, and the colon lengths were measured. (C) DAI was calculated. Mice treated with sulfasalazine (150 mg/kg) were used as positive controls. Data are represented as the mean (SEM) (n = 5) from triplicate experiments (^#^ *P* < 0.05 *vs.* control, * *P* < 0.05 *vs.* DSS alone).

The inhibitory effect of vanillic acid on the intestines in DSS-induced experimental colitis was evaluated. The physiological signs (weight loss, colon length, diarrhea, and occult/gross bleeding) after 5% DSS treatment were monitored for seven days, and their disease activity index (DAI) was calculated. As shown in Figure 1A and Figure 1B, all mice treated with DSS showed significant weight loss and colon shortening compared to the control group. However, we observed that groups administered with vanillic acid showed significant attenuation of DSS-induced body weight loss and colon shortening. In addition, DAI was remarkably inhibited in groups administered with vanillic acid as compared to the groups administered with DSS (Figure 1C). Sulfasalazine was used as a positive control in the present study.

### 2.2. The effect of vanillic acid on levels of IL-6 in DSS-induced colitis 

**Figure 2:**
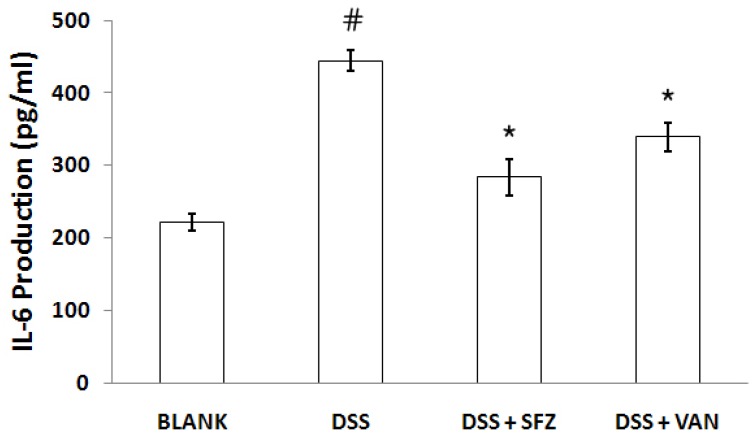
Effect of vanillic acid on the level of IL-6 in the plasma of DSS-treated mice. Experimental colitis in mice was induced by 7-day treatment with 5% DSS dissolved in the drinking water. Vanillic acid was administered orally at doses of 200 mg/kg once a day for seven days before DSS treatment. Mice treated with sulfasalazine (150 mg/kg) were used as positive controls. At the end of the experiment, blood samples were collected from all the mice and immediately centrifuged at 3,000 rpm for 15 min to separate the plasma. The levels of IL-6 in mouse plasma were evaluated by ELISA.

To investigate the effect of vanillic acid on plasma IL-6 levels in mice affected with colitis, enzyme-linked immunosorbent assay (ELISA) was performed. Blood samples were obtained from the mice at the end of the experiment. As shown in Figure 2, the levels of IL-6 were significantly increased in the DSS-treated group compared to those in the control group. However, administration of vanillic acid reduced these increased levels induced by DSS. 

### 2.3. The effect of vanillic acid on expression of COX-2 and NF-κB p65 in DSS-induced colitis

**Figure 3:**
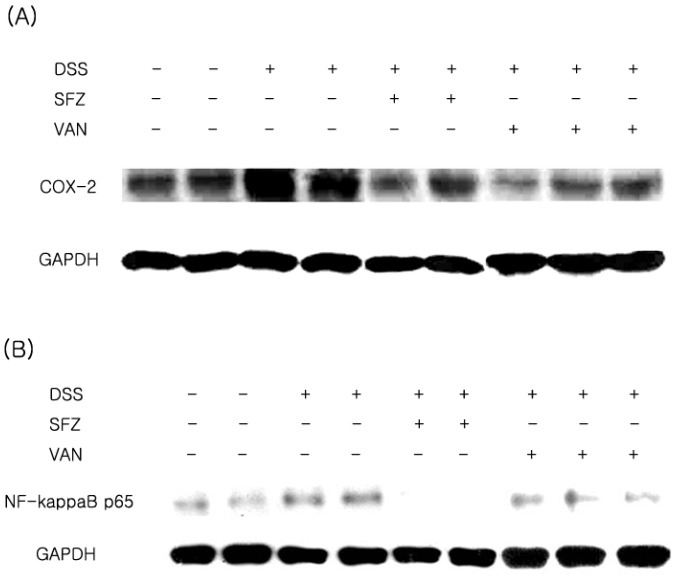
Effect of vanillic acid on the levels of COX-2 and NF-κB p65 in DSS-treated colon tissue. Experimental colitis was induced in mice by 7-day treatment with 5% DSS dissolved in drinking water. Vanillic acid was administered orally at doses of 200 mg/kg once a day for 7 days before DSS treatment. Mice treated with sulfasalazine (150 mg/kg) were used as positive controls. At the end of the experiment, the colon tissue was excised and homogenized. (A) The levels of COX-2 were evaluated by Western blot analysis. (B) The levels of NF-κB p65 were evaluated by Western blot analysis.

The effect of vanillic acid on the expression levels of COX-2 were evaluated by Western blot analysis. The expression of COX-2 was significantly increased in the colon tissues of DSS-treated mice compared to those in the control group. However, administration of vanillic acid reduced the expression of COX-2 induced by DSS (Figure 3A). Activation of NF-κB p65 is involved in colitis, and thus inhibition of NF-κB activation has been suggested as an anti-inflammatory strategy in colitis [13]. We examined whether vanillic acid regulated the activation of NF-κB p65 in the tissues affected by colitis. The activation of NF-κB p65 was significantly increased in the colon tissues of DSS-treated mice compared to those in the control group. Oral administration of vanillic acid significantly reduced the activation of NF-κB p65 induced in DSS-treated colon tissues (Figure 3B).

### 2.4. Discussion

UC is an idiopathic disease characterized by intestinal inflammation [20]. UC is commonly treated with glucocorticosteroids, sulfasalazine, and so on, however these drugs cause serious adverse effects such as hormonal disturbances, formation of peptic ulcers, liver dysfunction, and psychological problems. In the present study, we attempted to determine the effects of vanillic acid on DSS-induced colitis. UC is a chronic intestinal inflammatory condition manifested by symptoms including abdominal pain, weight loss, and bloody diarrhea [21,22,23]. The results of this study showed that mice treated with DSS had reduced body weight and colon length compared to the mice in the control group. However, treatment with vanillic acid reduced the weight loss and colon shortening caused by DSS. DAI, which was scored in terms of three major clinical signs (weight loss, diarrhea, and rectal bleeding), was remarkably reduced in mice treated with vanillic acid than in those not treated with vanillic acid. These results suggest that vanillic acid might effectively reduce the severity of the colitis symptoms caused by DSS. 

At a site of inflammation, the recruited cells are activated to release a host of inflammatory mediators, including tumor necrosis factor (TNF)-α, IL-6, and COX-2. These mediators contribute to the initiation and progression of the inflammatory process. The levels of inflammatory mediators, including COX-2 and IL-6, have been reported to be elevated in UC patients [7,8]. Therefore, the development of new biological therapies for inflammatory disease has generally focused on the blockade of members of the inflammatory cascade. Our findings showed that the levels of IL-6 and COX-2 were indeed higher in colon tissue of DSS-treated mice than in those not treated with DSS; however, these levels were reduced by treatment of vanillic acid. These results indicate that vanillic acid exerts an anti-inflammatory effect in UC via the regulation of COX-2 and IL-6 levels. We also observed that vanillic acid inhibited the IL-1β and TNF-α levels in the serum, but the reduction in these levels was not significant (data not shown). Therefore, we focused on the effect of vanillic acid on IL-6 levels. 

NF-κB is a transcription factor that is important for the activation of many inflammatory mediators, such as cytokines (e. g., TNF-α and IL-6) and COX-2. Studies have reported that macrophages in UC patient exhibit markedly increased NF-κB DNA-binding activity, which in turn is associated with the secretion of high levels of IL-1, IL-6, and TNF-α. In addition, the specific downregulation of NF-κB p65 led to considerable reduction in the production of proinflammatory cytokines, such as IL-1, IL-6, and TNF-α, by macrophages of IBD patients [24]. In this study, we observed that the NF-κB p65 activity was significantly higher in the colon tissues of DSS-treated mice compared to those in the control group. However, treatment with vanillic acid reduced this activity in the colon tissue of DSS-treated mice. On the basis of these findings, we think that vanillic acid inhibits NF-κB p65 activity, which leads to the suppression of the transcription of inflammatory mediators. Taken together, these findings suggest that vanillic acid exerts an inhibitory effect on the inflammatory response in UC through the regulation of NF-κB p65 activation. 

## 3. Experimental 

### 3.1. Animals and reagents 

Female BALB/c mice (6 weeks old) were obtained from Da-Mool Science (Taejeon, Korea). The mice were housed in a specific pathogen-free environment for at least one week to allow adaptation to the environment. All the animal studies were carried out in accordance with the regulations set by the Institutional Review Board of Wonkwang University (confirmation number: WKU09-081). DSS (mol wt: 36,000–50,000) was purchased from MP Biomedicals (Solon, OH, USA). Vanillic acid granules were purchased from Fluka (Buchs, Switzerland). Purified anti-mouse IL-6 and recombinant IL-6 were obtained from BD-Pharmingen (San Diego, CA, USA). The specific antibodies against COX-2 and β-actin were obtained from Santa Cruz Biotechnology (Santa Cruz, CA, USA). All chemical reagents were purchased from Sigma Co. (St. Louis, MO, USA).

### 3.2. Induction of colitis by DSS 

Acute colitis in mice was induced by providing drinking water ad libitum containing 5% (w/v) DSS for seven days. Mice were checked daily for loss of body weight, stool consistency, and the presence of gross bleeding. The mice were randomized into four groups: Group Ι (n = 5) was used as the control and was given only standard chow and water. Group ΙΙ (n = 5) received DSS in their drinking water. Group ΙΙΙ received DSS and vanillic acid (200 mg/kg) and Group ΙV received DSS and sulfasalazine (150 mg/kg). Vanillic acid and sulfasalazine diluted with water (200 μL) were orally administered once a day from day 0 of DSS treatment. The mice were killed by CO_2_ inhalation at the end of the 7-day DSS treatment, and their colon tissues were examined. 

### 3.3. DAI 

The activity of intestinal disease was assessed through manifestations comprising loss of weight, diarrhea accompanied with blood and mucus, and shortening of the colon [4]. In this study, DAI was determined by the method described by Murthy *et al.* [25], in terms of the scores of 3 major clinical signs (weight loss, diarrhea, and rectal bleeding). Loss of body weight was calculated as the difference between the initial and actual weight. Diarrhea was defined by the absence of fecal pellet formation in the colon and the presence of continuous fluid fecal material in the colon. The appearance of rectal bleeding was scored in terms of diarrhea with occult bleeding or gross rectal bleeding. DAI was calculated using the following formula: DAI = (weight loss score) + (diarrhea score) + (rectal bleeding score). The clinical parameters used are comprehensive functional measures that are analogous to the subjective clinical symptoms observed in human ulcerative colitis. This method of scoring has been validated in several studies.

### 3.4. Cytokine assay 

Blood samples were obtained from mice and immediately centrifuged at 3,000 rpm for 15 min to separate plasma. The levels of IL-6 were measured using a modified method of ELISA described elsewhere [26]. Ninety six-well plates were coated with a 100-μL aliquot of anti-mouse IL-6 monoclonal antibody at 1.0 μg/mL in phosphate-buffered saline (PBS) at pH 7.4 and incubated overnight at 4 °C. After additional washes, 100 μL of the sample or the IL-6 standards were added and incubated at 37 °C for 2 h. The wells were then washed, and 0.2 μg/mL of biotinylated anti-mouse IL-6 was added and incubated at 37 °C for 2 h. After washing the wells, avidin-peroxidase was added, and the plates were incubated for 30 min at 37 °C. The wells were washed again and 2,2’-azo-bis(3-ethylbenzthiazoline-6-sulfonic acid) (ABTS) substrate was added. Color development was measured at 405 nm by using an automated microplate ELISA reader. A standard curve was run for each assay plate by using recombinant IL-6 in serial dilutions. Protein concentration was measured using a bicinchoninic acid (BCA) protein assay kit (Sigma Co. St. Louis, MO, USA).

### 3.5. Western blot analysis 

Tissue samples of the distal colon (100 mg) were collected from all mice; the samples were then homogenized in 600 μL of lysis buffer (iNtRON Biotech, Korea), incubated for 30 min on ice, and centrifuged at 13,000 rpm for 5 min. The supernatants were transferred to a fresh tube, and their protein concentrations were determined using the BCA protein assay kit. Lysates (50 μg of protein) were separated by 10% SDS-PAGE and transferred to membranes (Amersham Pharmacia Biotech, Piscataway, NJ). The membrane was then blocked with 5% skim milk in PBS-Tween 20 for 1 h at room temperature and then incubated with the antibodies. After washing in PBS-Tween 20 three times, the blot was incubated with the secondary antibody for 1 h, and the antibody-specific proteins were visualized using an enhanced chemiluminesence detection system (Amersham Corp. Newark, NJ, USA) according to the recommendations of the manufacturer.

### 3.6. Statistical analysis 

The results are presented as the mean (SEM) of at least 3 experiments. The results were compared using independent *t*-tests and ANOVA with a Tukey post hoc test. A *P* value of < 0.05 was considered significant. 

## 4. Conclusions 

In summary, we have demonstrated that treatment with vanillic acid can significantly reduce the clinical signs of UC and levels of inflammatory mediators in a mouse model of DSS-induced colitis. On the basis of these results, we suggest that vanillic acid may be a useful therapeutic candidate for colitis. However, further studies must be performed to elucidate the precise mechanism of action of vanillic acid in the treatment of intestinal inflammatory disorders.
